# Can the ubiquitous power of mobile phones be used to improve health outcomes in developing countries?

**DOI:** 10.1186/1744-8603-2-9

**Published:** 2006-05-23

**Authors:** Warren A Kaplan

**Affiliations:** 1 Center for International Health and Development, Boston University School of Public Health, 85 E. Concord Street, Boston, MA 02118, USA

## Abstract

**Background:**

The ongoing policy debate about the value of communications technology in promoting development objectives is diverse. Some view computer/web/phone communications technology as insufficient to solve development problems while others view communications technology as assisting all sections of the population. This paper looks at evidence to support or refute the idea that fixed and mobile telephones is, or could be, an effective healthcare intervention in developing countries.

**Methods:**

A Web-based and library database search was undertaken including the following databases: MEDLINE, CINAHL, (nursing & allied health), Evidence Based Medicine (EBM), POPLINE, BIOSIS, and Web of Science, AIDSearch (MEDLINE AIDS/HIV Subset, AIDSTRIALS & AIDSDRUGS) databases.

**Results:**

Evidence can be found to both support and refute the proposition that fixed and mobile telephones is, or could be, an effective healthcare intervention in developing countries. It is difficult to generalize because of the different outcome measurements and the small number of controlled studies. There is almost no literature on using mobile telephones as a healthcare intervention for HIV, TB, malaria, and chronic conditions in developing countries. Clinical outcomes are rarely measured. Convincing evidence regarding the overall cost-effectiveness of mobile phone " telemedicine" is still limited and good-quality studies are rare. Evidence of the cost effectiveness of such interventions to improve adherence to medicines is also quite weak.

**Conclusion:**

The developed world model of personal ownership of a phone may not be appropriate to the developing world in which shared mobile telephone use is important. Sharing may be a serious drawback to use of mobile telephones as a healthcare intervention in terms of stigma and privacy, but its magnitude is unknown. One advantage, however, of telephones with respect to adherence to medicine in chronic care models is its ability to create a multi-way interaction between patient and provider(s) and thus facilitate the dynamic nature of this relationship. Regulatory reforms required for proper operation of basic and value-added telecommunications services are a priority if mobile telecommunications are to be used for healthcare initiatives.

## Background

There is an ongoing, broad policy debate about the value of communications technology in promoting development objectives. The literature is diverse in its opinions. Some view computer/web/phone communications technology as merely providing a 'quick fix' for solving development problems that must be solved with comprehensive policies cutting across all sectors. Similarly, some view communications policy as increasing social gradients, in large part because of the existence of knowledge and information barriers, lack of skilled human capital and lack of funds for modernization [1]. Those who disagree about these negative positions argue that harnessing communications technology will benefit all sections of the population, will disseminate information, open opportunities for women. They point to Africa and the Arab States, in which the poor as well as the uneducated have been able to access this technology in public facilities, shared services and other innovative strategies [2,3].

Within the context of this broad policy debate on the value of information technology in developing countries, there is a specific issue that deserves attention. Are mobile telephones a potentially useful intervention to deliver healthcare, including healthcare information, in developing countries? Mobile telephone subscriptions have been growing rapidly since the 1980s in both developing and developed countries. Subscriptions to fixed telephones have also grown, but in many parts of the world growth has been at a slower rate than cellular. The demand for mobile phones exists beyond reducing the waiting list for traditional wire-line phones [1].

In 2002, mobile subscribers overtook fixed line subscribers worldwide and this occurred across geographic regions, socio-demographic criteria (gender, income, age) or economic criteria such as gross domestic product (GDP) per capita [4]. In much of sub-Saharan Africa, there are more mobile phones than fixed-line phones [5] and the use of mobile phones in many Asian countries is on the rise.

A more formal definition of a healthcare "intervention" in the present context is the following: it is an intentional activity that comes between persons or events for the specific purpose of modifying some health-related outcome or act. Thus, for the purposes of this discussion, an "intervention" has the sense of an intentional use of mobile phones to achieve a specific purpose. The functioning of the telecommunications market, by itself, is not considered an "intervention." For instance, although the mere presence of a mobile telephone in a village may enable communication with healthcare providers and lessen isolation in case of emergency, this is not considered an intervention as defined above. However, use of subsidized phones or airtime or more sophisticated applications using exiting mobile phone platforms for the express purpose of supporting or altering one or more health outcomes would be considered an "intervention".

"Telemedicine" encompasses many different communication modalities and is not a single technology. It includes video and other conferencing, transmission of computed tomography (CT) images, and computer-assisted or Web-based provider-patient communication systems. Various uses of telephones have contributed to this repertoire of "telemedicine", defined as the delivery of health care and sharing of medical knowledge over a distance using telecommunications (1). In this regard, the predominant modality has been fixed telephones, in combination with enhancements such as computer-automated, telephone follow-up and counseling, telephone reminders, interactive telephone systems, after-hours telephone access, and telephone screening. See, e.g., [7-10]. There is continuing interest from academics, clinicians and policy makers about the value of these interventions to improve health outcomes and quality of life [5-8]. The term "e-health", originally used as an industry and marketing term, has also found its way into the scientific literature and may be supplanting "telemedicine" as the latest term for a very dynamic subject matter. One may briefly define "e-health" as both a structure and as a way of thinking about the integration of health services and information using the Internet and related technologies.

Part I is a brief literature review of the uses of fixed telephones and mobile telephones as a healthcare intervention for management of a variety of diseases. What is the evidence that telephones in general, and mobile phones in particular, can be effective as a healthcare intervention in developing countries? The Discussion (Part II) summarizes the issues on both sides, that might persuade or dissuade, a potential stakeholder in a developing country from initiating healthcare interventions using mobile phones. Use of mobile telephones as a healthcare intervention in developing countries has tremendous, but as yet untapped, potential due to technical as well as financial and regulatory barriers.

## Methods

A Web-based and library database search for intervention studies (as defined above) in developing countries was initiated using the following terms: "mobile", "SMS", "cell phone", "telephone", "telecommunications", "policy", "wireless", "telemedicine", in various combinations with "healthcare", "health", "adherence", "HIV", "tuberculosis", "intervention", "compliance", "developing country", "Africa", "Asia". Searches included MEDLINE, CINAHL, (nursing & allied health), Evidence Based Medicine (EBM), POPLINE, BIOSIS, and Web of Science, AIDSearch (MEDLINE AIDS/HIV Subset, AIDSTRIALS & AIDSDRUGS) databases. Only included those references were used where data could be extracted or, at a minimum, where the abstract was available. Thus, references in difficult-to- find journals and/or without an abstract are not included. Reviews of "telemedicine" generally (which include telephonic interventions) can be found in [7,9,11-13].

## Results

### Literature review

The relative lack of information for developing countries is striking. It is obvious, however, that most studies found are in wealthy nations comprising members of the Organization for Economic Cooperation and Development (OECD). Of the 3870 total participants in various fixed telephone interventions (Table 1), fully 94% (n = 3640) were in the United States. For mobile phone interventions (Table 2), of the 852 participants, 88% (n = 753) were from Europe, Japan or Korea but the reasons for this relative geographic distinction between fixed and mobile are obscure.

**Table 1:** Using Telephones as a Healthcare Intervention: Fixed Phones

**Country**	**Indication/Disease**	**Intervention**	**Results**	**Reference/Comments**
Newfoundland Canada	Diabetes outcomes	To assess whether modem link from patient at home to hospital improves diabetes control. RCT: transmission of blood data via modem; N = 42. Patients in "telephone group" performed five blood glucose determinations/day twice/week and transferred data via phone once/week. Control group brought results in to clinic every 6 wk. "Telephone" group counselled every week via telephone to adjust insulin and food intake Duration = 12 weeks.	In treatment group, HbA1c improved from 0.106 to 0.092 (13.20%). The control group improved from 0.112 to 0.102 (8.9%). No significant change in weight, random blood glucose, or insulin.	[14]
United States	Breast cancer: mammography	RCT: in-person v. telephone v. no mammography counselling. N = 1098. Duration = 4 weeks	Compared to no counselling, telephone counselling was more than twice as effective at increasing mammography adherence, and in-person counselling resulted in almost three times the mammography adherence.	[15]
United States	Tuberculosis: adherence to medication	Observational videophone Directly Observed Therapy, Short Course (DOTS) program v. standard DOTS. Two way links between home and health department. N = 6. Duration = 24 months	During 304 video- observed treatment doses, adherence was 95%, and patient acceptance of the technology was excellent. Adherence on standard DOT was 97.5%. A total of 8830 driving miles were avoided/288 travel hours	[16] "In selected cases, the use of videophone technology can maintain a high level of adherence to DOT in a cost-effective manner"
United States	Various indications: patient outcomes	RCT: follow-up phone call by a pharmacist 2 days after discharge from hospital. N = 221. Data collected on patient satisfaction and outcomes. Duration = 7 months	Phone call group more satisfied with discharge medication instructions (86% vs. 61%, P = 0.007). Fewer patients from phone group returned to ER within 30 days (10% phone call vs. 24% no phone call, P = 0.005).	[17]
United States	Hypertension :adherence to medication	RCT: usual medical care v. computer-controlled telephone system in addition to usual medical care to promote adherence. N = 267 Duration = 6 months	Mean antihypertensive medication adherence improved 17.7% for telephone system users and 11.7% for controls (P = .03). Mean DBP decreased 5.2 mm Hg in users compared to 0.8 mm Hg in controls (P = .02).	[18]
United States	Hypercholesterolemia: maintenance of change	RCT: Computer assisted telephone: two calls/month for six months v. no calls to maintain initial cholesterol change and provide feedback for patients completing a diet and behavioral cholesterol reduction program. N = 115 Duration = 6 months	Neither group fully maintained initial cholesterol reductions	[19]
United States	Diabetes outcomes	Observational study: Voice-interactive telephone system (daily self-measured glucose levels or hypoglycemic symptoms). N = 184 Duration = 12 months	Yearly prevalence of diabetes-related crises or hypoglycemia decreased from 3% of total calls to 2% (P < 0.05), with a concomitant statistically significant decrease in Type 2 diabetic HbA1c from 9.7, (SD = 1.03) to 8.6, (SD = 1.54, p = .03)	[20]
United States	Attendance at adolescent clinic	CT: Telephone reminder 1 day before clinic appointment v. no reminder. N = 703 Duration = 11 months	Attendance rate (65.2%) in intervention group was increased by 47.8% over control	[21]
United States	Diabetes outcomes	CT: Type 1 diabetes N = 10 Duration = 6 months	Proactive telephone intervention delivered by psychology undergraduates (15-min telephone intervention weekly for 3 months and biweekly for 3 additional months) Intervention group showed 1.2% drop in HbA1c; control group an increase of 0.8%., p < .05	[22]
United States	Depression outcomes	RCT: usual care v. telephone care management (feedback to patients/algorithm based intervention) v. telephone care management plus treatment recommendations/practice support N = 613	Compared with usual care, the practice telephone support intervention led to lower mean depression scores (2.59, P = .008). Compared with usual care, feedback only had no significant effect on treatment received or patient outcomes. Patients receiving feedback plus care management had a higher probability of both receiving at least moderate doses of antidepressants (odds ratio 1.99, 95% confidence interval 1.23 to 3.22) and a lower probability of major depression at follow up (OR = 0.46, 0.24 to 0.86).	[23]
United States	Immunization rates	Computer-generated telephoned reminders v. control intervention to raise the rates of on-time immunization among preschool-age children in two public clinics in Atlanta, GA.	Intervention group households had faster vaccinations (adjusted OR = 2.12: 1.01, 4.46) but the overall effect of the intervention on immunization levels appeared to be minimal (crude relative risk = 1.07, 95 percent confidence interval = 0.78, 1.46). Only 80 percent of children in both groups were members of a household with a telephone number listed in clinic records.	[24]
United States	Hypertension adherence to medication	RCT: Nurse administered- intervention via telephone bimonthly v. usual care for hypertension. N = 294 Duration = 2 years	Blood pressure (BP) control not yet reported. Patients with nurse intervention had a greater increase in confidence of their BP management following hypertension treatment than the usual care group.	[25]
United States	HIV	Cross sectional study within clinical trial: Compare and contrast three different methods for measuring self reported ARV adherence: nurse rating, self report and recall phone interview. N = 35 adolescents	Little agreement between phone calls, clinical nurse rating and self report regarding the level of adherence.	[26] Phone calls were time and labor intensive. "... not recommended as part of regular clinical practice".
Various	Immunization Rates	Cochrane Review	All types of reminders were effective (postcards, letters, telephone or autodialer calls), with telephone being the most effective but most costly. Effect on rates for childhood vaccinations (OR = 2.02, 95% CI = 1.49,2.72), for childhood influenza vaccinations (OR = 4.19, 95% CI = 2.07,8.49), for adult pneumococcus or tetanus (OR = 5.14, 95%CI = 1.21, 21.8), and for adult influenza vaccinations (OR = 2.29, 95%CI = 1.69, 3.10).	[27]

RCT = randomised controlled trial; CT = controlled trial

**Table 2:** Using Telephones as a Healthcare Intervention: Mobile/Wireless Communication

**Country**	**Indication/Disease**	**Intervention**	**Results**	**Reference/Comments**
Denmark	Asthma	Observational study: SMS Text: asthma "diary". Patients received 4 SMS messages/day, including a medication reminder, a request to enter peak flow, data on sleep loss, and medication dosage. Participants were asked to reply to a minimum of 3 of the messages per day. Diary inputs were collected in a database. N = 12. Duration = 2 months	SMS collection of asthma diary data is "feasible" half the participants reported more than about two thirds of the requested diary data.	[28] "The combination of SMS data collection and a traditional Web page for data display and system customization may be a better and more usable tool for patients than the use of Web-based asthma diaries which suffer from high attrition rates"
Italy	Quality of Life Questionnaire	Feasibility study. Questionnaire delivered as display on mobile phone, answered with keypad. N = 97. Duration = 12 days	Fifty six (58%) attempted the questionnaire, and all of these 56 completed it. patients who refused to participate were older, had fewer years of education and were less familiar with new communications technology (mobile phone calls, mobile phone SMS, internet, email).	[29]
United States	HIV	Feasibility study: Automated two-way messaging system to improve ARV adherence. N = 25. 17,440 messages and 14,677 replies (84%). Duration = 208 days (median)	"...high satisfaction with the messaging system ... it helped with medication adherence." Participants reported missing one or more doses on 36% of 743 queries.	[30]
Tenerife	Diabetes	Feasibility study: PC Web browser or a mobile phone capable of working with the WAP protocol to transmit blood chemistry data to clinic. N = 12. Duration = 9 months	Patients used system every 2.0 days and doctors reviewed data every 4.0 days Seventy five percent expressed a preference for sending their data via the mobile phone SMS	[31]
Hong Kong	Various	Wireless Application Protocol (WAP)-based telemedicine system for patient-monitoring	WAP 1.1 phone used at 1800 MHz by circuit-switched data (CSD) to connect to the content server through a WAP gateway, which was provided by a mobile phone service provider in Hong Kong. "Data were successfully retrieved from the database and displayed on the WAP phone. "	[32]
Japan	Body weight monitoring	Feasibility study: Mail function of the mobile phone for use in maintaining body weight reduction as the achievement target. N = 136. Duration = 4 months Subjects informed on body weight reduction knowledge and practice once/day via mailing	" [T]endency for reduced body weight was found in 63 (46%) of 136 adults. Average body weights were significantly reduced (P < 0.001) from 73.2 kg to 71.1 kg (males), and from 58.8 kg to 57.6 kg (females)	[33]
Korea	Diabetes	Pre-post study. Internet/SMS texting. N = 185. Participants sent self-measured blood glucose levels, medication, dosages, meal, and exercise to their provider. Laboratory tests including lipid profiles and glycated hemoglobin (HbA1c), and a survey of satisfaction before and after study period. Duration = 3 months.	The mean HbA1c improved from 7.5 +/- 1.5 to 7.0 +/- 1.1% after using the management program (P = 0.003).	[34]
Spain	Hypertension	RCT: Comparative, controlled, multicenter, randomized cluster study. SMS texting to patients re: compliance. Control group received usual interventions; intervention group received messages and reminders sent to their mobile phones 2 days per week. N = 104. Duration = 4 months	No effect on compliance. 85.1% (CI, 74.9%-95.3%) in the control group and 84.4% in the intervention group (CI, 70.7%–95.3%) (P = NS). NO effect on control of hypertension	[35]
United States	Hospice patients	Feasibility study: alphanumeric paging system as a memory enhancer for various therapeutic regimens	Compliance rose from a mean of 56 percent to 96 percent when the system was used.	[36] Unclear from abstract which regimens were affected
Scotland	Asthma	Observational study. N = 30. Mobile phone text message service consisting of daily reminders to use an inhaler, health education tips, and safety messages.	There were no adverse safety events, and the service was technically reliable. "Compliance with using an inhaler may have favorably changed in response to the service."	[37] Only anecdotal evidence to support the conclusion
United States	Smoking cessation	Web and cell phone technologies to deliver a smoking-cessation intervention. N = 46.	At 6-week follow-up, 43% had made at least one 24-hour attempt to quit, and 22% were quit – based on a 7-day prevalence criterion.	[38] Duration of intervention unknown
Croatia	Asthma	RCT: GSM mobile telephone SMS texting study All subjects received asthma education, self-management plan, and standard treatment. All measured PEF three times daily and kept a symptom diary. In the study group, therapy was adjusted weekly by an asthma specialist according to PEF values received daily via SMS from the patients N = 16. Duration = 16 weeks.	There was NO significant difference between the groups in absolute PEF. NO significant difference between the groups in daily consumption of inhaled medicine, forced vital capacity, or compliance. Additional cost of follow-up by SMS was Euros 1.67/patient/week (equivalent to approximately $1.30 per 1 Euro), and SMS transmission required 11.5 minutes. Controls had significantly higher scores for cough (1.85 +/- 0.43 vs. 1.42 +/- 0.28, p < 0.05) and night symptoms (1.22 +/- 0.23 vs. 0.85 +/- 0.32, p < 0.05).	[39] Study group of 40 patients is needed to achieve the power of 80% within the 95% confidence interval.
Spain	Cardiovascular disease	Feasibility study. Patients provided with portable recording equipment and a cellular phone that supported data transmission [electrocardiogram (EKG)] and wireless application protocol (WAP) N = 89. Mean length of participation = 50.1 days.	A total of 2168 EKGs (mean duration transmission = 2 min/30 s; network errors < 0.1%) and 4011 short messages (none lost, in 95% of cases 30 s < delay < 1 min) were transmitted.	[40]
Finland	Diabetes	Non-randomized, controlled study: Transmission of glucose values by cellular phone in the treatment of type 1 diabetic patients. SMS message sent to the patients 1/week giving instructions v. controls receiving standard treatment without instructions 1/week. N = 100 consecutive patients/controls Duration = 1 year	"The phone system was not associated with overall improvement in HbA1c, probably due to the patients' low measurement activity."	[41] "Sophisticated electronic systems are not beneficial to all patients, but should be restricted to those having high motivation to use them."
Spain	Vaccination rates Hepatitis A and B Whether reminder of the next vaccine dose sent by SMS increase compliance with hepatitis A + B and hepatitis A vaccination schedule.	SMS sent to the vaccinee's mobile phone. Trained health-care workers entered the data into a computer to generate text messages reminding vaccinees of their scheduled doses.	For the second hepatitis A + B dose, compliance in the study group (Message Groups) was slightly improved (88.4%: 83–92%) over two separate controls 80.7%: 76–84%) and 77.2%: 73–80%). For hepatitis A vaccine, compliance rates for the second dose were 27.7%:24–32%) and improved over controls 16.4%:14.4–18.6%) and 13.2%: 11.6–14.9)	[42]
South Africa	Tuberculosis	SMS text messaging to improve DOTS using a modified Medication Event Monitoring System ^® ^(MEMs) bottle cap that sends a signal to a mobile service provider.	Adherence not measured	[43–44]
South Africa	HIV	Cell Life^® ^project, has developed software and data management systems that let clinic workers use their mobile phones to monitor patients' treatment. Information collected is sent to a central database	Data published at the Civil Engineering Department of The University of Cape Town.	[45]

RCT = randomised controlled trial; CT = controlled trial

As this review was not intended to be exhaustive, it is difficult to generalize because of the different outcome measurements and the small number of controlled studies. The majority of reports are "pilot" or "feasibility" studies. A subset of Tables 1 and 2 is presented below as Table 3 for diabetes and hypertension- two of the conditions where there is useful information with respect to outcome measurements.

**Table 3:** Effect of Telephone Interventions on Outcomes for selected Chronic Conditions

**Condition (sample size)**	**Outcome Measure**	**Change in Outcome Measure**	**Reference**	**Comments**
Diabetes (42)	HbA1c	13.2% decrease (intervention) v. 8.9% decrease control)	[14]	Duration = 3 months
Diabetes (142)	Prevalence of hypoglycemia HbA1c	Decrease from 3% to 2% Decrease from 9.7 to 8.6	[20]	Change in HbA1c statistically significant Duration = 1 year
Diabetes (10)	HbA1c	1.2% decrease (intervention) v. 0.8% increase (control)	[22]	Duration = 6 months
Diabetes (185)	HbA1c	Decrease in 0.5 units	[34]	Duration = 3 months
Diabetes (100)	HbA1c	No change	[41]	Duration = 1 year
Tuberculosis (6)	Adherence to medication	Similar adherence outcomes between intervention and control	[16]	Duration = 2 years
Hypertension (267)	Adherence	17.7% adherence improvement (intervention) v. 11.7% control	[18]	Duration = 6 months
Hypertension (104)	Adherence	No effect on compliance	[35]	Duration = 4 months
Asthma (16)	Lung capacity Compliance	No effect on absolute lung peak expiratory flow (PEF) and medicine compliance	[39]	Duration = 4 months

Aside from recent work in South Africa [43-45], there is almost no literature on using mobile telephones as a healthcare intervention for chronic, non-communicable diseases such as cardiovascular disease, diabetes, depression, and for chronic, communicable diseases such as HIV and TB. Even in developed countries, except for certain diabetes studies, clinical outcomes are rarely measured. There is almost nothing known about how such technology could be scaled up beyond the pilot stage. Moreover, the cost effectiveness of telephonic interventions is not known. A recent systematic review [46] of telemedicine (including other interventions besides telephonic ones and largely confined to developed countries) found that only a small percentage of eligible studies (7/24 (29%)) even attempted to explore the level of utilization that would be needed for telemedicine services to compare favorably with traditionally organized health care. No studies that were reviewed addressed this question in sufficient detail to adequately answer it. These authors concluded that there " ... is no good evidence that telemedicine is a cost effective means of delivering health care." [46] Evidence regarding the effectiveness or cost effectiveness of mobile telephones in particular as a telemedicine intervention is therefore still limited [46,47]. This is a weak evidence base upon which to develop policy or allocate resources.

We note that for any intervention to be "cost effective" as a means to enhance adherence to medicines, it would have to be effective in reducing the burden of illness associated with non-adherence at an optimal level of resource use. A recent review on this subject [47] was not able to make definitive conclusions about the cost-effectiveness of such interventions to enhance adherence to medicines " ... due to the heterogeneity of the studies found and incomplete reporting of results." In this recent cost- effectiveness review [47], forty-three studies were reviewed and 41 were conducted in OECD countries, the remaining two being in Malawi (malaria prophylaxis compliance [48]) and Botswana (home-based v. hospital-based TB care [49]). Difficult policy decisions are being made all the time about "rationing", i.e., the allocation of finite healthcare resources [50], and the cost-effectiveness of mobile phone technology as a healthcare intervention will become part of these decisions, if they are not already.

## Discussion

Not withstanding the apparent paucity of evidence in developing countries that is more than anecdotal, certain functional and structural properties of mobile phones may make them attractive to use as a healthcare intervention.

### 1. Attractions of using mobile telephones as a healthcare intervention

#### Low start-up cost

Living in resource-poor environments is not a barrier to use of wireless for several cultural and economic reasons. There appears to be a lower threshold of access to cell phones [51]. That is, there is evidence that the existence of a so-called "digital divide" along the socio-economic gradient is less pronounced in mobile phones than in other communication technologies such as the Internet [52]. Furthermore, mobile (i.e., wireless) costs less to rollout over large areas than does a fixed phone line and mobile networks can be built faster than fixed lines [4,5]. The social value of a mobile phone is highly valued even in resource-poor areas.

Households in developing countries may spend up to 2% of their monthly expenses on communication [5]. From an economic viewpoint, mobile phones have a shorter payback on investment compared to land lines, in large part because the scalability of mobile is greater compared to other infrastructure investments. Functionally, mobile phones are easier to use for people with lower level of skills than those needed for computers or the Internet, both of which usually require land lines.

#### User friendly- SMS

Pricing policies may enhance certain mobile uses, in particular use of Short Messages System (SMS) text. SMS texting is rapidly growing and is boosted in some countries such as the Philippines as a text message is less expensive than a phone call. SMS provides low bandwidth digital messaging between users and has surprised some observers by its success. Even as early as 1999–2000, the number of SMS messages in the United Kingdom grew from 159 million to 1.42 billion. In 2003, the average user in the Philippines sent 2,300 messages, making it the world's most avid texting nation. SMS is a part in almost all marketing campaigns, advocacy, and entertainment. In fact, SMS is influential enough in the Philippines that several local dotcoms like Chikka Messenger [53] and Bidshot [54] now fully utilize SMS for their services. There are a number of practical, and not very surprising, reasons for using SMS. It cost less than voice messaging and it can reach people whose phones are switched off. SMS messaging is silent which means that messages can be sent and received in places where it may not be practical to have a conversation.

#### Forms of payment and market potential

The standard way of paying for a mobile phone service in the United States and Europe is on the basis of a minimum use of a certain time period per month for a year. Potential customers have to provide proof of a regular income, sign a contract, and have a bank account and a permanent address. Since the vast majority of people in developing countries likely do not have any of these, mobile service providers use a prepayment system. This involves buying cards which provide phone time from five minutes to an hour. Customers can use the credit as they like over a period of weeks, and so keep control over their spending and enjoy a very cheap phone service. Prepaid cards are widely available in local stores. Once the pre-paid "outgoing call budget" has been exceeded, many persons will continue to use the mobile phone but will only receive calls. In 1998, three years after the first prepaid mobile phone scheme was launched, 40 million people were using it – about 13 per cent of the world's mobile users. In South Africa, half of all subscribers chose prepayment. In Zambia at present, all mobile phone systems use use this scheme. Prepaid telephone calling cards allow people to get money together to buy one cellular phone among them, purchase prepaid cards, and then control phone usage.

Given the sharing of mobile phones in many places and the popularity of pre-paid phone cards, evaluating the profitability of mobile telecommunications in many developing countries by considering calls made **from **the phone and not calls **received **is probably inappropriate [55,56]. Indeed, although the global average percentage of prepaid mobile subscribers out of total mobile subscribers in 2004 was about 46%, this ranged from 31% in Asia, 45% in the Americas, 62% in Europe to 87% in Africa [57].

### 2. Barriers to use of mobile telephones as a healthcare intervention

#### Cost issues

The penetration of mobile phones in large parts of the developing world notwithstanding, mobile access is more expensive than fixed line access since one is paying for "coverage" rather than connection to a specific location [4,58]. Makers of mobile handsets make their profits selling high-end units to consumers in developed countries so profit margins may have to be much lower in emerging markets such as Africa [56]. In most countries in the developing world, it is still expensive to buy a handset and novel strategies to improve connectivity have arisen, such as the practice of sharing mobile phones in communities. Compared to the average income of its inhabitants, the cost of a one minute outgoing call on a mobile network in most non-European/U.S. countries is arguably quite expensive, ranging from $0.50 in Brazil, to $1.00 in Senegal to $1.30 in Nigeria [57]. Lack of electricity will be a problem although this can be overcome in clever ways, e.g., one person takes village's cell phones to have them all charged at once [5].

#### Information carrying capacity

The low bandwidth of mobile phones leads to a lack of structure and nuance in content. SMS text messages are limited to 160 characters. Although SMS messaging is silent, the restriction on structure means that it may be difficult to carry on a potentially complex real-time interaction between patient and provider. Further, costs of data transmitted over mobile phone are greater than voice costs. Extensive use of transmitting data using mobile phones in developing countries has not been demonstrated [5,55].

#### Language and illiteracy

Pervasive illiteracy may be the rate-limiting step on use of SMS text messaging [4] and the combination of illiteracy and indigenous languages may have dramatic effects on the use of SMS messaging. The implications of this will extend to use of text messaging to convey health information. For example, in the UK, the ratio of the number of outgoing voice calls made to the number of outgoing SMS messages sent is 0.6:1. In South Africa as a whole, the ratio was 3:1 for pre-paid phones and in the rural communities surveyed by Vodafone, the average ratio was a remarkable 13:1 [5]. In Ndebe, a rural community in South Africa, the ratio was 17:1, but when one considers this in the context of a community in which education is not universal, the data are more understandable [5]. We note that if new communication technologies are introduced slowly, then SMS text messaging will not be replaced anytime soon but illiteracy will clearly impact its use. The development of voice recognition-mobile phone applications would also be useful in countries with high levels of illiteracy but this is a third generation (3G) application and does not seem likely to impact many resource-poor countries in the near future. Nonetheless, illiteracy does not have to be an insurmountable barrier. The CyberTracker project [59] allows mostly non-literate San people of the Kalahari in Southern Africa to transfer their knowledge about migratory movements of wild animals by giving them handheld portable computers with a touch-sensitive screen. In conjunction with signs and symbols and an attached GPS, field data is rapidly collected. Such modalities are possible using mobile phones enabling Java technology.

The mobile phone (e.g., wireless) industry has done very well selling low bandwidth "pipes" for connectivity, and it appears determined to increase the content available on mobile phones [60]. The 3G systems will provide considerably higher bandwidth than current phones, and will include images, Internet access, and videos. This bandwidth is universally touted as a way to provide Internet access, and in particular to sell content to users. SMS messages can leave a record, whereas a telephone conversation will not. The ability to extract old SMS text may be important for privacy of healthcare information for TB or HIV-infected persons where the threat of being stigmatized is present.

## Conclusion

### 1. There is not enough evidence to support or refute the claim that mobile phones "work" as a healthcare intervention

With regard to Tables 1 and 2, perhaps we should not be surprised that the effects of telephone interventions on various clinical and other outcomes are mixed. To conclude that such interventions probably work **some **of the time is a trivial response. More significantly, and particularly with respect to improving medication adherence in important chronic non-communicable conditions that are increasingly prevalent in less developed countries (hypertension, diabetes, depression), any realistic intervention to improve adherence must be both dynamic and sustainable over time as patients' lives and circumstances will change. Adherence interventions must be temporally flexible and creative to track changes in the patients' relationship to the healthcare system. Indeed, such interventions as summarized in Tables 1 and 2 might in principle be effective **most **of the time provided we can understand how to give the appropriate message in a way that becomes an integral part of the recipients' life. This is clearly true whether or not phones are used as the intervention. This long-term contextual view of adherence to medicines is particularly germane to the chronic conditions mentioned previously. A health-related message must be understood consistently over time and be culturally and socially appropriate to the indication and to the real-time needs of the patient. This is a daunting challenge for whatever medium is used. A recent review [61] of the varied health-related uses of SMS applications suggests that it " deliver [s] both efficiency savings and improvements in the health of individuals and public health." However, many of these uses have not yet been subjected to clinical trials and none have been systematically extended on a large scale. The overall lack of well designed, randomized clinical trials with economic evaluation to confirm or refute clinical and economic benefits with mobile phone/healthcare interventions is an evidence gap that should be addressed in a systematic way.

The physical components of a telephone, i.e., the handset or headset and the network, are not isolated but are part of an entire system that includes pricing plans and other incentives which can provide leverage employed by healthcare professionals and policymakers. Notwithstanding any impact on health outcomes by the message itself, the effect of mobile phones, the particular payment plan and related components. i.e., the medium itself, on delivery of the "intervention" is not well understood either. Indeed, the medium that delivers an intervention may have a neutral, positive, or even negative impact on the health intervention it is delivering. This aspect of the debate about use of telecommunications as a healthcare intervention has hardly been addressed at all, in any environment.

### 2. A developed world model of mobile phones may not be appropriate in developing countries

**Figure 1:**
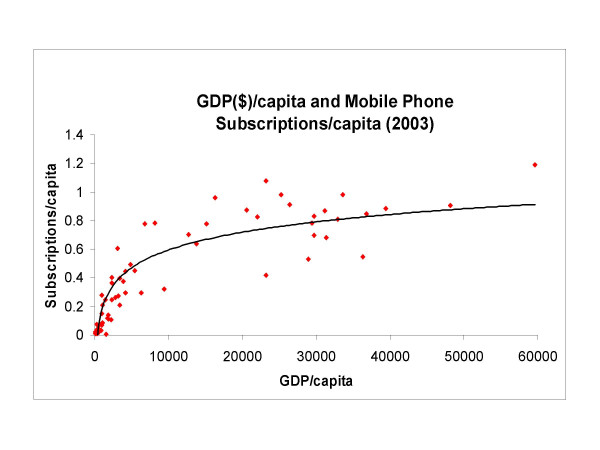
The Relationship of GDP/capita (US$-2003) and Mobile phone subscriptions/capita (2003) for Various Countries. Data obtained directly from reference [62] as reproduced in additional File 1.xls.

Inter-country comparisons of aggregate statistics for 73 countries derived from the International Telecommunications Union [62] are shown in Figure 1, below and in additional File 1: Spreadsheet.xls of summary statistics of GDP per capita and mobile subscriptions per capita for various countries.

In Figure 1, the relationship between GDP/capita and mobile phone subscriptions per capita suggests that small changes in "wealth" will result in large changes in mobile phone penetration in poorer countries at GDP/capita less than about $3–4,000. Whether or not this inference really holds for resource-poor countries that lie at the lower end of this graph is an open question. The non-linear nature of Figure 1 also suggests that income has less of an effect on mobile phone penetration per capita in the more affluent countries. It is worth noting that the nature of Figure 1 is similar to the relationship between "wealth" and health indicators such as life expectancy. The ramifications of this latter relationship are still subject to continuing debate. It is possible that the health of individuals in a society also depends on the degree of income inequality in that society and that the effect of distribution of income on health, and possibly on many other things including mobile phone penetration, is more important than absolute income. Aggregate-level analyses of "developed" and "developing" countries will not illuminate issues about determinants of individual health, or mobile-phone use as related to health. The question as to whether computer/web/phone communications technology can solve development/health problems should be shifted from a discussion about 'developing vs. developed" countries to whether use of telecommunications, and mobile telephones in particular, in healthcare is appropriate to the specific national and local context.

In Africa, mobile penetration rates are low by developed country standards but use of pre-paid calling cards and the informal sharing of mobile phones between people all increase accessibility, even in rural communities. The impact of mobile extends well beyond what might be suggested by measuring the aggregate number of subscriptions. Shared use in some locations could be an important constraint if mobile phones are to be used to convey health information since two-way communication in a shared system is difficult as a non-owning user can make outgoing calls but cannot receive spontaneous calls [4]. SMS text messages, if not deleted, can be observed by subsequent users. These informal arrangements that extend the reach of telecommunications beyond the individual user seem very powerful. Policy debates on information technology policy generally and health policy in particular are not sufficiently informed by evidence of this type [5].

### 3. Creating a sustainable, large-scale mobile phone/healthcare model requires agreement among different stakeholders with different agendas

The work summarized in Tables 1 and 2 are almost invariably small, academic pilot or feasibility studies. A major unresolved issue when approached from the point of view of "who is doing the intervention" relates to whether these studies can be scaled-up in the community and whether they can have an impact on individual and, ultimately, on public health. Table 4 summarizes the different perspectives of some of the major stakeholders who might be expected to use mobile phone technology in a large-scale health intervention.

**Table 4:** Stakeholders' Positions regarding Mobile Phones as a Healthcare Intervention

	**Patient**	**Healthcare Provider**	**Mobile Phone Company**
Focus	Individual	Individual/Care Group	Potential Clients
Outcome	Absence/amelioration of disease	Absence/amelioration of disease/reduce cost of care	Product sales
Motivation	Well being through treatment	Professionalism through treatment. Profit through cost containment	Profit through new sales, new products, marketing user acceptance

Patients are looking at an intervention using telecommunications broadly, and mobile telephones in particular, to eliminate or at least ameliorate suffering and reduce their financial burden during the illness and healing process. With respect to aspects of healthcare counselling, some patients may prefer face-to-face contact rather than phone or text message contact. For some persons, communication of almost any type using SMS messages will lack nuance and individual "tailoring" so that synchronous, real-time voice communication between patient and healthcare provider will be preferred. Real-time communication can clearly be realized using mobile phone technology. A consideration with respect to asynchronous communication, i.e., with a time lag between sending and receiving, is that such communication may have to be secured or otherwise encrypted, especially with shared and/or stolen mobile phones.

From the viewpoint of a patient with TB or HIV or epilepsy, the ease of use of mobile devices could be a potential problem since, unless encrypted in some way, an e-mail/text message opened because of a theft or viewed inadvertently will increase the risk of being stigmatized. It is not clear if this issue is important in actual practice. "Privacy" can be seen as an aspect of security – one in which trade-offs between the interests of one group and another can become particularly clear [63]. Security services (e.g. that based on digital signatures) probably do not come without transaction costs to the end-user as well as society since supportive law would need to be implemented in many countries. Nonetheless, in mobile infrastructure in developing countries, privacy/security and authentication services can be based on certificates and secret keys implemented in SIM (Subscriber Identity Module) cards. Here the patients and healthcare professionals may sign and prove digitally, and if needed, encrypt all their communications. This is a subject well beyond the scope of this paper but see, for example [64].

Healthcare providers are also looking for treatment that will eliminate or at least ameliorate suffering and improve communication of health-related issues between themselves and patients. Providers in managed care settings utilizing telecommunication/mobile structure as an intervention nonetheless might share the same concern, albeit based in easing their own financial burden and improving their bottom line. From this viewpoint, voice counselling may be time and money- intensive so providers may actually prefer automated interactions. Although a provider's first priority might be to proactively transmit information via mobile phone to the patient (i.e., "We notice that your blood sugar has gotten low... do this..."), the ability of this to make a clinical difference will be a function of whether the patient can understand the information and act upon it. This is therefore a function of the mobile phone context, i.e., its intrusiveness, timing, quality, clarity.

It is worth noting that with respect to using mobile phones to monitor diagnostic indices, any chemical, biological or physical marker must be easily determined and easily sent via the mobile phone. Blood glucose, spirometry, adherence (e.g., number of cigarettes/pills), blood pressure, weight, physical activity, mental state, side effects can all be transferred with relative ease. For HIV there is no simple diagnostic useful in this context as a patient cannot now simply phone in their CD4 or viral load count. Weight loss and known side effects are more likely markers for "wireless" monitoring of HIV status. The great potential advantage of mobile phone technology in managing chronic conditions is that it can collect small amounts of data rapidly, efficiently and with minimum intrusion. A healthcare intervention that requires communication of relatively simple information (e.g. weight or a spirometry result or a blood glucose value) may be preferable to content that demands more sophisticated modalities like video. Even with the relatively simple interventions under review here, the mobile phone company must be aware of possibly unique legal issues relating to security, privacy authentication, theft of identity, liability for harm due to unauthorized/negligent transmission of health information and the like [64].

From a business point of view, mobile telephone companies make their profit in the private sector. They are only likely to invest in such technology in the public research sector for reasons of – for want of a better term- "corporate responsibility". Clearly, however, the more realistic priority in scaling-up mobile phone infrastructure to support a phone-based healthcare intervention will be to keep their existing clients and attract new ones. Monitoring the cost of the content (the message) as opposed to mere connectivity (the medium) is important. An additional consideration is their attempt to manage their way through a changing regulatory environment, especially with state-owned telecom networks [52,65,66]. Creating a sustainable business model among the stakeholders, as well as insurers and pharmacists will be needed and is a challenge A supportive legal, governmental and business infrastructure for such a model is no less a challenge in a developed country.

New modalities such as broadband access technologies (e.g. WiMAX, Flash-OFDM, VoIP and so on) are being created all the time. Within these infrastructures, not only data (e.g. web, e-mail), but also voice over internet (VoIP) services will be widely possible in many places. With these new wireless access technologies, transmission speeds of 500–1000 kilobit/s, even higher, are possible. When framed in the present context, the question of whether or not these are suitable modalities for improving health outcomes, must be informed by the particular social and behavioral health context at several levels, i.e., country-level down to patient-level.

The larger debate about communications technology as a barrier or spur to development may not be resolved for some time. The communications and services infrastructure to support large-scale use of telecommunications as a health intervention exist in some parts of Africa and in much of Asia. At present, one would hope that healthcare applications such as accessing medical self care, receiving medication adherence reminders (e.g., all the applications used in developed countries), facilitating case management of chronic conditions (e.g., diabetes, TB) are more suitable for the majority of the poor in developing countries [55,56,61,67], than receiving mortgage information or buying concert tickets.

Notwithstanding the fact that large-scale supportive infrastructure exists, a top priority goal for all governments should be to (re)-align the regulatory and pricing policy of the telecommunications sector with health policy goals. Use of various information technologies (including mobile telephones) to less developed countries and communities has been ongoing for some time, mostly via the many specific initiatives, led by communities, development, donor and business organizations. Evidence on the effectiveness of these initiatives with particular regard to their use as healthcare interventions is mostly in the form of anecdotal material. More rigorous evidence is needed for drawing conclusions.

○ The developed world model of personal ownership of a phone may not be appropriate, and may even be irrelevant, to the developing world where telephones are often shared.

○ Convincing evidence regarding the cost-effectiveness of mobile phones as a " telemedicine" intervention is limited and good-quality studies are rare in less developed countries.

○ Evidence of the cost effectiveness of fixed or mobile telephones as such an intervention to **improve adherence **to medicines was difficult to identify. Given the rapid expansion of chronic disease management (TB, HIV, non-communicable chronic conditions) in less developed countries, the ability of mobile telephone interventions to improve long-term adherence to medicines in chronic disease is unknown but could be of major benefit. Such interventions must be part of a repertoire of interventions to be used in a changing way over the lifetime of a patient. One advantage of telephones to manage chronic disease is its ability to create a two-way interaction between patient and provider(s) and thus facilitate the dynamic nature of the relationship and accompanying interventions.

○ A framework for debate among telecommunications, development and public heath experts about the use and value of mobile phones as health intervention in developing countries will have to account for the different primary perspectives of the relevant stakeholders, the value-added of each stakeholder in a sustainable business model, as well as the context-specific nature of information technology systems in general. For a mobile telephone system to be successful, whether or not as a healthcare intervention, it has been shown that the local context is understood.

○ Regulatory reforms required for proper operation of basic and value-added telecommunications services are a priority if mobile telecommunications are to be used for healthcare initiatives.

## Competing interests

The author(s) declare that they have no competing interests.

## Supplementary Material

Additional file 1 Gross Domestic Product per capita (US $) and Mobile Phone Subscriptions per capita for selected countries. Data obtained directly from Reference [62]. U.S. dollar figures obtained by applying average annual exchange rates from the International Monetary Fund (IMF) to the national currency for that year. Where such rates were unavailable, a World Bank or United Nations conversion rate was used. Click here for file
